# Last mile at the center of immunization delivery

**DOI:** 10.3389/fepid.2026.1913390

**Published:** 2026-08-25

**Authors:** Grace Umutesi, Sara Rendell, Ruanne V. Barnabas

**Affiliations:** 1 Department of Global Health, University of Washington, Seattle, WA, United States; 2 Division of Infectious Diseases, Mass General Brigham, Boston, MA, United States; 3 Harvard Medical School, Boston, MA, United States

**Keywords:** equity, global health, immunization, last mile delivery, vaccine-preventable diseases

## Abstract

While advances in immunization led to major reductions in vaccine-preventable diseases in the past four decades, large gaps remain in the delivery of immunization services. In this perspective, we use illustrative examples to discuss gaps in global immunization and outline opportunities for evidence generation and intervention design to transform the last mile delivery of immunization services in global health. To address last mile gaps, technical limitations in global health surveillance infrastructure that result in imprecise estimates and overlapping obstacles that complicate immunization service delivery for selected groups need to be addressed. Additionally, leveraging pro-equity strategies from the community level to the global level and across phases of translational health research could accelerate the equitable impact of immunization innovations on population health outcomes.

## Introduction

Over the past four decades, advances in immunization drove major reductions in vaccine-preventable diseases. Maternal tetanus immunization programs reduced neonatal tetanus incidence by 89% and mortality from tetanus by 84%, with 47 high-priority countries achieving elimination (1, 2). Universal hepatitis B vaccination at birth lowered chronic infection in children under five from approximately 5% to 1% (3). Human papillomavirus (HPV) vaccines were proven to be highly effective in preventing high-risk HPV infection which has markedly reduced HPV-related disease (4). Since its inception in 2,000, Gavi, the vaccine alliance, has helped vaccinate 1.2 billion children and prevent more than 20.6 million deaths from vaccine-preventable diseases, and enabled 18 countries to establish fully sustainable immunization systems (5). However, large gaps in immunization delivery leave millions sick with and dying from vaccine preventable diseases (2, 6, 7).

Interventions that seek to address the “last mile” gap in the delivery of immunization services must solve several overlapping problems (8, 9). First, technical limitations in global disease surveillance infrastructure result in imprecise estimates that guide and define success of vaccination programs because certain populations are not included in these estimates. Additionally, delays in national policy adoption, gaps in funding for vaccination programs and limited vaccine product availability shape which vaccines may reach the last mile. Finally, significant obstacles to immunization service delivery result in repeated situations in which immunization strategies do not effectively improve coverage for selected groups even when policies, funds and products are available. In this perspective, we use selected examples to discuss these overlapping gaps in global immunization and outline the opportunities for evidence generation and intervention design across phases of translational health research to transform the last mile delivery of immunization services in global health.

## Imprecise estimation of populations at the last mile

Standard case definitions, a system of active or passive case reporting and operational surveillance systems are necessary to capture cases and estimate disease burden. To say that many are missing from coverage denominators is a truism. Populations facing serial displacement and de-documentation (including remote communities, mobile populations, stigmatized groups, people displaced by conflict) are likely to be under-enumerated and therefore difficult to account for in immunization planning and coverage indicators (10). For example, one quarter of children in the world are born into areas affected by conflict and with constrained health resources; these children account for 50% of all children having received zero doses of recommended vaccines (11, 12). In 2024, 60 major measles outbreaks coincided with an estimated 6 million children in Nigeria, Ethiopia and the Democratic Republic of Congo who did not receive a single dose of a measles-containing vaccine (11, 12). These missed doses result from displacement, conflict, and resource constraint.

Accounting for under-documented populations is not merely a technical challenge with a neutral solution. For stigmatized populations and displaced groups, documentation can carry the risk of exposure to harmful state surveillance, including immigration enforcement, and loss of services that may be contingent on non-disclosure (13, 14). To remain under-enumerated may therefore reflect protective navigation of risk rather than merely a data-system failure (15, 16). Additionally, the challenges of under-enumeration will continue to intensify as ecological niches for pathogens expand to impact an increasingly mobile global population exposed to climate-related disaster, conflict, and governance failures (17).

## Gaps in vaccine introduction and delivery at the last mile

Even when vaccine strategies exist, gaps in national adoption and financing determine which populations are offered the vaccines. Thus, delays in vaccine introduction illustrate an adjacent problem to denominator exclusion. For example, more than 90% of pneumonia-related deaths occur in LMICs (2, 8). *Streptococcus pneumoniae* causes pneumonia, meningitis, and endocarditis, all of which are prevented by the pneumococcal conjugate vaccine (PCV). Global coverage for the PCV remains low (8). Factors that enable successful introduction of PCV include high coverage of common childhood vaccines, supportive governments, functional technical advisory bodies, and Gavi support (8). Collective bargaining strategies, for example through Gavi's Advance Market Commitment or UNICEF's pooled procurement strategy, combined with procurement from lower cost suppliers can lower the cost of PCV per-dose to approximately 2.5 to 3.0 USD per dose, which is up to 10 times lower than prices paid by non-Gavi eligible countries and up to 50 times less than the private market prices in high income countries, which can be 132 USD per dose (18, 19). Uniform access to such strategies is needed to make delivery of this life-saving vaccine affordable to countries (8). Contexts in which these factors are absent result in a situation where it would be unreasonable to expect adaptation of existing strategies to be effective.

Furthermore, chickenpox, primary infection with varicella zoster virus (VZV), causes an estimated 4.2 million severe complications and 4,200 deaths across the globe each year (20). After primary infection, reactivation, known as herpes zoster, is common and 19.1 million cases of reactivation are expected by 2030, with more than one in five of these cases having complications (21). Countries that introduced childhood varicella vaccination, saw an 80 to 95% reduction in incidence of chickenpox and its complications, including death (22). Still to date, fewer than one fourth of WHO member states have introduced varicella immunization, and this life-saving vaccine has yet to be routinely provided in any country in Africa (23). With low coverage of the varicella vaccine (<80%), there are concerns about shift of disease to older groups with higher rates of complications (22). The table below summarizes key aspects of four vaccine-preventable infections to illustrate distinct aspects of last-mile equity challenges, specifically entrenched under-vaccination and under-enumeration (measles), price and access dynamics (pneumococcal conjugate vaccine), evolving access and dosing debates (VZV), and novel vaccine strategies for diseases with highest burden in low income settings (malaria). Table 1 includes estimates of respective vaccine efficacy, burden of the disease, and vaccination coverage. Taken together, the selected examples above suggest we are, in practice, endlessly deferring the last mile. As the factors that lead to loss of human lives from coverage denominators are expected to increase, the indicators that define our failures will be imprecise. These examples call for strategies that innovate beyond mere acceptance that some populations could be left behind.

**Table 1:** Global vaccination gaps.

Selected examples	Vaccine efficacy	Global Disease Burden	Global coverage
Measles Virus	Two-dose series Clinical efficacy: 97%	Incidence (cases per million): 155.8 in Africa; 162.2 in Europe; 62.2 globally (24)	15.6 million children have not received the 1st dose (25)
			Global coverage for 2nd dose is 76% (26)
*Streptococcus pneumoniae*	Three-dose series Clinical efficacy: ∼75.0% against invasive pneumococcal disease (27)	50% of global pneumococcal deaths in 2015 occurred in India, Nigeria, DRC and Pakistan (28) Global incidence of meningitis 2.51 million cases per year; 18.1% of meningitis deaths attributed to S pneumoniae (6)	Global coverage has plateaued at ∼67% (23)
			32 WHO member states have not introduced pneumococcal vaccine (26)
Varicella Zoster Virus	Primary varicella vaccine • Two-dose series • Clinical efficacy 92.4% (two doses), 74.7% (single dose) (29)	Primary infection: • >4 million severe complications • >4,000 deaths per year (20) Reactivation: • >19 million cases expected by 2030 with complications in >20% (21)	Only 47 of 192^a^ countries have introduced the varicella vaccine (no African countries) (23)
*Plasmodium* species (Malaria)	Three-dose primary series followed by booster RTS,S AS01E: Clinical efficacy against severe malaria ∼58% (30)R21/Matrix-M: Clinical efficacy against malaria ∼68–75% (31)	282 million cases globally in 2024 resulting in 610,000 deaths, 75% of deaths were in children under 5 years old (32)	56 of 80 malaria endemic WHO member states have introduced the vaccine (23) 3rd dose coverage ranges from 22 to 70%

a Following the 2026 withdrawals of the USA and Argentina, WHO membership stands at 192 states rather than 194.

## Proposed pro-equity solutions

Surveillance weaknesses need to be addressed to close structural gaps that result in sub-optimal delivery of immunization services. This can be achieved by prioritizing pro-equity strategies that seek to address measurement issues and incorporate them in implementation plan starting from the planning and design phase. Approaches to address measurement gaps should focus on improving the availability of disaggregated data by leveraging Geographic Information Systems (GIS) Mapping to identify underserved communities, community-based monitoring, targeted surveys and microplanning to capture groups that are not captured at the macro-level (33).

Approaches that have previously been successful in contributing to equitable delivery of vaccination services should not be considered proven fixes but rather testable strategies to be investigated and adapted in settings where they are relevant or appropriate. For example, Gavi has already begun to focus on engagement strategies that enable countries to have more agency over how Gavi supports local vaccine programs and the health systems that deliver them (34). This transition provides opportunities for developing and testing approaches that address local barriers to improve equity. Specifically, community oriented vaccine communication and service delivery can increase vaccine confidence and coverage (15, 35). In settings with migrants and mobile populations, co-delivering human and animal vaccination among pastoral communities, integrating nutrition and immunization services, leveraging mobile posts in refugee communities to conduct vaccination campaigns, and collaborating with community leaders contributes to increasing coverage or uptake of routine immunization services (36). Leveraging community health care workers and community-oriented approaches through home visits and community engagement has shown potential to increase vaccine uptake or coverage in these communities (15). In Colombia, for example, the announcement of temporary protection status to allow refugee and migrant communities to access free vaccination services led to a relative increase in COVID-19 vaccination uptake by 26.2% over just four months (13).

Additionally, a systematic review that included twelve studies focused on improving vaccination uptake or coverage among people living with disabilities reported that the most studies (11/12, 92%) focused on individuals with physical disability or chronic health conditions. Only two studies were conducted in LMICs or LICs, highlighting the need for research in these settings (37). While interventions that improve immunization information management systems (e.g., electronic medical record alerts, reminders, and recalls) may strengthen provider-based delivery strategies, innovative approaches should also focus on community-oriented strategies that enhance accessibility and meet recipients where they are. To reach communities that are frequently left out for multiple reasons, culturally tailored services delivered through accessible and community-centered approach should be co-designed and implemented in partnership with communities to ensure that interventions reflect their realities and address accessibility challenges from their perspective (35).

Rural communities are often prioritized in efforts to improve immunization coverage. However, communities living in urban slums and other disadvantaged urban areas must also be placed at the center of these efforts. Approaches that have contributed to improved coverage in these settings include mobile clinics, school-based vaccination programs and decentralized planning that leveraged community-oriented health workers and information system to strengthen service delivery. These approaches, however, should be adapted to local contexts and account for the needs of multiple priority groups, including people living with disabilities in urban slums and migrant communities. They should also consider how intersecting structural and social factors influence the availability of and access to essential health services which ultimately shape health outcomes.

Generating new evidence on more efficient and equitable approaches to immunization service delivery should be prioritized, particularly in a time of shifting funding priorities, as the delivery context and global ecosystem continue to evolve. Countries commitment to sustainably finance vaccination was reflected by more than US$ 250 million contribution made in 2024 towards their own immunization programs but this progress was derailed by recent changes in funding (38). Specifically, changes in bilateral health assistance such as the dismantling of USAID that played a direct and indirect role supporting immunization service delivery complicate the implementation of pro-equity strategies since more financial resources would be required to digitalize health records, conduct GIS mapping or in-dept community engagement activities (39).

One example is the HPV vaccination. Access to HPV vaccines is inequitable and HPV-related malignancy remains a leading cause of cancer-related deaths in women worldwide, with 88% of women who die of cervical cancer living in a LMIC (4, 40). The immunologic and clinical efficacy of fewer doses (1 and 2 doses) compared to the initial three-dose schedule was evaluated after the vaccine was licensed in 2006 in efforts to improve uptake. In 2022, nearly two decades after the vaccine was licensed as a three-dose schedule, the WHO recommended that countries adopt a single-dose schedule for the targeted age group to improve coverage, citing evidence of comparable efficacy and durability of protection to the two-dose schedule (4). A single-dose schedule offers several advantages that can advance vaccine equity, health equity and gender equity (41). Requiring fewer doses, reduces the logistical complexity and costs associated with HPV vaccine delivery, thereby improving access for populations who need the vaccine the most. Furthermore, a single-dose schedule, enables vaccination in settings where repeated contact with communities is challenging or not feasible. Since evidence on the efficacy of a single-dose schedule for people living with HIV is limited, the WHO recommendation retained a two-dose schedule recommendation for immunocompromised individuals including people living with HIV.

Implementing a single-dose schedule frees resources (including vaccine, supplies, and personnel) that could be used to expand HPV vaccination beyond girls aged 9–14 years old, who are the primary target group in most settings. This could enable the expansion of the HPV vaccination program to: 1) older girls and young women; 2) women-living with HIV, who are at increased risk of acquiring persistent HPV infections leading to cervical cancer; and 3) boys and men, to reduce the transmission and burden of HPV-related malignancy (41). Since the licensing of HPV vaccines, implementation research has made important contributions to the adaptation of HPV vaccination schedules by generating evidence from diverse settings on the effectiveness, health and economic impact, and stakeholders perspectives on reduced-dose schedules. These insights have catalyzed progress toward cervical cancer elimination. Similar approaches could be considered early in the development and implementation of trials or novel technologies to ensure that insights from high-burden settings are incorporated at the outset. Doing so may accelerate the translation of appropriate evidence-based intervention to achieve the greatest public health impact. Table 2 summarizes selected examples of gaps existing in the delivery of immunization services and proposed pro-equity approaches to address these gaps.

**Table 2:** Examples of existing gaps in immunization service delivery and proposed pro-equity solutions.

Gap	Phase of Translational Health Research	Proposed solution
Surveillance and measurement gaps	Translation to community (Population-level outcomes research)	Triangulate data across sources to identify community gaps Leverage digital immunization registries with unique identifiers and disaggregated data Use GIS mapping, targeted survey, community monitoring to track progress and adjust delivery plans Engage in microplanning to enable community ownership and better reach missed communities
Equitable and efficient approaches to delivering immunization services	Translation to practice (Clinical implementation)	Evaluate the efficacy of existing vaccination recommendations established during vaccine licensing (schedule, dosage, boasters, etc.)
	Translation to community (Population-level outcomes research)	Evaluate delivery strategies to improve outcomes Prioritize immunization delivery to most vulnerable groups
Approaches to improve vaccination coverage among people living with disabilities	Translation to community (Population-level outcomes research)	Co-design and co-implement community-centered strategies to improve access and meet recipients where they are Account for intersecting structural and social factors that influence access and availability of services when designing or adapting interventions
Approaches to reach mobile populations, displaced populations and communities living in urban slums		

## Conclusion

Incredible progress has been made over the past four decades in the delivery of immunization services. Yet this same period has revealed how people routinely fall outside of surveillance and beyond the practical limits of accepted delivery models. Climate change, conflict and constraints on health infrastructure will intensify immunization gaps for an increasingly mobile population. Equity, providing what is needed to close gaps, therefore must be at the center of design principles, dictating how evidence is generated, which innovations are prioritized, where programs start, and how success is measured.

Today, we must build systems that place those most often missed at the center of our delivery strategies. We must ask, who are constantly slipping beyond the reach of key interventions such as vaccination so we can design and evaluate strategies that place these populations first. Immunization agendas should invest in community-partnered delivery from the outset, flexible service platforms within governance that reduces exclusionary status. Implementation research should be pursued early to enable streamlined deliveries, appropriate adaptation to align with setting-specific realities, rather than being retrofitted to them.

## Data Availability

The original contributions presented in the study are included in the article/Supplementary Material, further inquiries can be directed to the corresponding author/s.
